# Neuroprotection and immunomodulation by dimethyl fumarate and a heterologous fibrin biopolymer after ventral root avulsion and reimplantation

**DOI:** 10.1590/1678-9199-JVATITD-2019-0093

**Published:** 2020-05-20

**Authors:** Paula R. G. Kempe, Gabriela Bortolança Chiarotto, Benedito Barraviera, Rui Seabra Ferreira, Alexandre L. R. de Oliveira

**Affiliations:** 1 Laboratory of Nerve Regeneration, University of Campinas (UNICAMP), Campinas, SP, Brazil.; 2 Center for the Study of Venoms and Venomous Animals (CEVAP), São Paulo State University (UNESP), Botucatu, SP, Brazil.

**Keywords:** Ventral root avulsion, Dimethyl-fumarate, Fibrin sealant, Neuroprotection, Immunomodulation

## Abstract

**Background:**

Ventral root avulsion (VRA) is an experimental approach in which there is an abrupt separation of the motor roots from the surface of the spinal cord. As a result, most of the axotomized motoneurons degenerate by the second week after injury, and the significant loss of synapses and increased glial reaction triggers a chronic inflammatory state. Pharmacological treatment associated with root reimplantation is thought to overcome the degenerative effects of VRA. Therefore, treatment with dimethyl fumarate (DMF), a drug with neuroprotective and immunomodulatory effects, in combination with a heterologous fibrin sealant/biopolymer (FS), a biological glue, may improve the regenerative response.

**Methods:**

Adult female Lewis rats were subjected to VRA of L4-L6 roots followed by reimplantation and daily treatment with DMF for four weeks. Survival times were evaluated 1, 4 or 12 weeks after surgery. Neuronal survival assessed by Nissl staining, glial reactivity (anti-GFAP for astrocytes and anti-Iba-1 for microglia) and synapse preservation (anti-VGLUT1 for glutamatergic inputs and anti-GAD65 for GABAergic inputs) evaluated by immunofluorescence, gene expression (pro- and anti-inflammatory molecules) and motor function recovery were measured.

**Results:**

Treatment with DMF at a dose of 15 mg/kg was found to be neuroprotective and immunomodulatory because it preserved motoneurons and synapses and decreased astrogliosis and microglial reactions, as well as downregulated the expression of pro-inflammatory gene transcripts.

**Conclusion:**

The pharmacological benefit was further enhanced when associated with root reimplantation with FS, in which animals recovered at least 50% of motor function, showing the efficacy of employing multiple regenerative approaches following spinal cord root injury.

## Background

Brachial and lumbosacral plexopathies result from exceptionally violent trauma, such as high-speed car and motorcycle accidents and falls from heights. This type of injury is usually overshadowed by other injuries, such as hemorrhage, shock and other complications [1, 2]. Diagnose of neurologic damage is limited and prognosis is poor; therefore, the patient usually develops persistent motor loss and severe pain. Causes for the lack of consistent recovery are multifactorial, including motoneuron degeneration due to proximal axotomy, vascular failure, gliosis, and chronic inflammation. The distance between the motoneuron cell body and the target muscle is also a great challenge to overcome [3, 4].

Ventral root avulsion (VRA) in adult rats is well known to cause extensive motoneuron degeneration, leading to the loss of approximately 80% of all axotomized neurons during the first two weeks after lesion [5-8]. VRA also causes several changes in the spinal cord environment, such as significant loss of synapses [9, 10] and cytotoxic and degenerative responses [11-13]. However, the surviving neurons show strong intrinsic regenerative potential. Increased glial responses influence gray matter plasticity by regulating ion diffusion in pre- and postsynaptic elements, increasing antioxidant activity [14-18]. Excitatory glutamatergic inputs are pruned from the motoneuron cell body, contributing to an inhibitory state over the alpha motoneurons [10, 19, 20], thereby suppressing action potentials to favor cell repair [21].

Pharmacological rescue of lesioned motoneurons precedes the anatomical reconstruction of the Central Nervous System (CNS)/Peripheral Nervous System (PNS) interface. In this context, dimethyl fumarate (DMF) has shown promising neuroprotection, anti-inflammatory and antioxidative properties [22-25]. DMF is a drug that promotes gene transcription of antioxidant and detoxifying enzymes [26, 27], leading to protection and the reestablishment of cellular homeostasis [28]. In addition, DMF modulates the immune system, reducing pro-inflammatory cytokines and diminishing inflammatory responses [22, 29-32]. DMF is also capable of improving the morphological preservation of myelin, axons and neurons, prolonging their survival and viability, and reducing spinal cord inflammation [26, 33-36]. In fact, DMF is currently used for immunomodulation of multiple sclerosis and has been approved by the FDA.

Considering the above-mentioned evidence, different strategies have been proposed to enhance neuronal survival and axonal regeneration either by reducing neuronal death or by controlling gliosis and its deleterious effects [5, 8, 37, 38]. Among these approaches, root reimplantation to the spinal cord stands out. In the first days after injury, root reimplantation results in neuronal survival and allows axonal regeneration, with consequent functional recovery [11, 39, 40]. This functional recovery is believed to be due to the secretion of neuroprotective factors from glial cells that stimulate the survival of axotomized neurons, combined with guided axonal growth towards the periphery [11, 41-43].

Root reimplantation can be performed with the use of fibrin sealant (FS), a biological adhesive that provides sufficient mechanical support at the interface of the CNS and PNS [44, 45]. The heterologous FS derived from snake venom (i.e., *Crotalus durissus terrificus*) is bioactive, does not induce cytotoxic or other adverse reactions [46-48] and decreases the chance of transmission of infectious diseases like AIDS and syphilis by not using human blood in its composition [44]. Additionally, FS produced similar results to conventional commercial glue, providing adequate adhesion and repair of rootlets after lesioning in rodents [8].

Therefore, we hypothesized that the association of pharmacological treatment with DMF and root reimplantation through the use of FS derived from snake venom may promote neuroprotection, preservation and recovery of motor function, which may in turn offer a new therapeutic option for patients with spinal cord injuries.

## Methods

### Experimental groups and treatments

**Figure 1.**
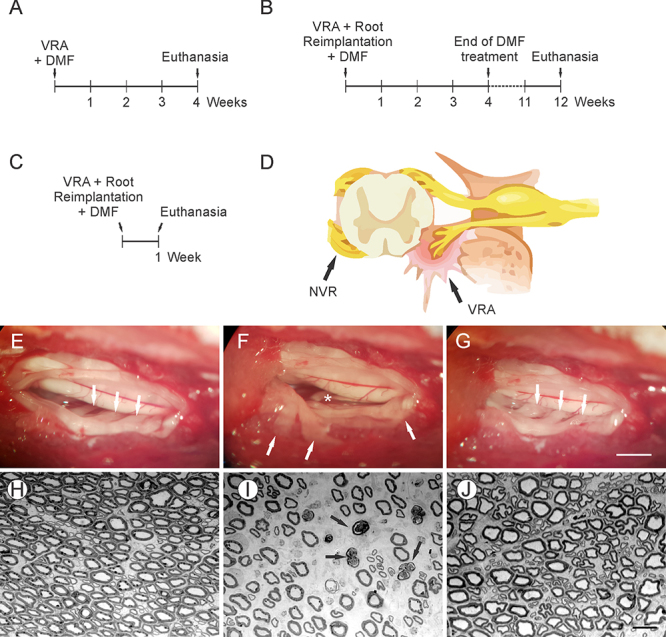
Schematic diagrams showing the experimental design with time points of surgical and pharmacological treatments throughout the experiment. **(** A **)** DMF treatment began immediately after VRA surgery and continued for 4 weeks. **(** B **)** Immediate reimplantation surgery using FS after VRA and subsequent DMF treatment for 4 weeks but maintained for a total of 12 weeks, which is the time point at which motor recovery is observable. **(** C **)** Animals destined for RT-PCR analysis were treated with DMF for 1 week (endpoint of analysis). **(** D **)** Schematic diagram shows a transverse view of the spinal cord highlighting the ventral (motor) root avulsion. **(** E **)** Rat spinal cord without lesion; arrows indicate ventral roots at their original site. **(** F **)** Ventral root avulsion of L4, L5 and L6 segments from the lumbar intumescence; arrows point the avulsed ventral roots which were placed laterally to the spinal cord to prevent any regeneration from the injured spinal segment. **(** G **)** Reimplanted roots at their original site; arrows point roots replaced to their original site; note the swelling of the roots. **(** H **)** Transverse section of a normal peroneal nerve. **(** I **)** Transverse section of the peroneal nerve after VRA; arrows point to axons undergoing degeneration; observe the spaces between axons due to the degenerative processes following VRA. **(** J **)** Transverse section of the peroneal nerve after VRA and reimplantation with FS; note close to normal compactness of endoneural environment, indicating that reimplantation led to successful axonal regeneration. NVR: normal ventral root; VRA: ventral root avulsion, DMF: dimethyl fumarate. (E-F) Scale bar = 1mm. (H-J) Scale bar = 10 μm.

Seventy-nine adult female Lewis rats (180-200 g) were housed with food and water *ad libitum* in a controlled environment with a 12/12-h light/dark cycle. All experiments were approved by the Committee for Ethical Use of Animals from University of Campinas (CEUA/UNICAMP, protocol number 4500-1/2017). Dimethyl fumarate (DMF, 242926, Sigma-Aldrich) was diluted in 0.08% methylcellulose (Sigma-Aldrich) saline. Methylcellulose alone was administered to the vehicle control group. Animals were randomly allocated into 3 different experimental settings, (n = 5/group/technique):

- Analysis of DMF dose-response effectiveness: 25 animals were submitted to VRA without root reimplantation and orally treated daily for 4 weeks with DMF (0, 7.5, 15, 30 and 45 mg/kg; gavage); the collected specimens were used for morphological and immunofluorescence evaluation (Fig. 1A).
- Analysis of motor functional recovery: 24 animals were submitted to VRA with or without root reimplantation with fibrin sealant, orally treated daily for 4 weeks with the most effective dose of DMF (15 mg/kg; gavage) and kept for another 8 weeks, totaling 12 weeks post-surgery; the collected specimens were used for morphological and immunofluorescence evaluation and motor function recovery evaluation (Fig. 1B).
- Analysis of gene transcripts levels at the acute phase post injury and repair of the motor roots: 5 animals with no lesion and 25 animals submitted to VRA with or without reimplantation were used for RT-qPCR analysis and orally treated daily for 7 days with DMF (15 and 30 mg/kg; gavage) (Fig. 1C).

### Ventral root avulsion (VRA)

The animals were anesthetized with a combination of xylazine (Anasedan, 10 mg/kg, Sespo Indústria e Comércio Ltda, Paulínia, SP, Brazil) and ketamine (Dopalen, 90 mg/kg, Sespo Indústria e Comércio Ltda, Paulínia, SP, Brazil). A dorsal incision, parallel to the spine, was performed in the upper lumbar/lower thoracic region. The paravertebral musculature of the spine was moved to expose the lower thoracic and upper lumbar vertebrae. A laminectomy of approximately three vertebrae was performed to expose the lumbar intumescence. The dural sac was opened through a longitudinal incision, and after dissection of the denticulate ligament, the ventral roots were moved and followed carefully until the respective rootlets could be detected and avulsed. Unilateral avulsion was performed by removal of the ventral rootlets at the L4, L5 and L6 spinal segments with fine forceps (N^o^ 4). After the surgical procedures, the musculature, fascia and skin were sutured in layers. The animals were kept on postoperative observation until recovery; analgesic was used for 3 consecutive days (tramadol hydrochloride - 5 mg/kg; gavage). 

### Ventral root repair and fibrin sealant

The FS derived from snake venom was composed of three separate solutions, which were homogenized immediately before use and applied at the lesion site in a final volume of 6 μL with the aid of a 10 μL pipette: (1) fibrinogen derived from bubaline blood (3 μL), (2) calcium chloride (2 μL), and (3) a thrombin-like enriched fraction (1 μL) [8, 44, 49-51]. Reimplantation in the appropriate animal groups was performed immediately after VRA. Thus, the avulsed roots were replaced at the exact point of lesion (Fig. 1G), and the first two components of the FS were applied. The third component was then added for polymerization. The reimplanted roots were then gently pulled from the spinal cord, and the stability of the fixation was observed to evaluate the success of the repair. The FS derived from snake venom was kindly supplied by the Center for the Study of Venoms and Venomous Animals (CEVAP - São Paulo), given that the constituents and instructions for use are patented (registration numbers BR1020140114327 and BR1020140114360).

### Functional analysis

For motor function recovery, we evaluated gait recovery using the CatWalk system (CatWalk, Noldus Inc., Wageningen, Netherlands). In this setup, each animal crosses a walkway with an illuminated glass floor. A green LED illuminates the long edge of the floor so that the light only highlights the places where the paws touch the glass surface. Through the illumination of the footprints, the plantar surfaces were captured by a high-speed video camera (Fujinon DF6H-1B) equipped with a wide-angle lens (8.5 mm, Fujicon Corp., China) positioned underneath the walkway. The paw prints were automatically recorded and classified by the software. Data were collected in quadruplicate (4 runs in each day tested). Preoperative data were recorded twice to provide a baseline, and postoperative data were collected every 6 days for 12 weeks. The peroneal functional index (PFI) was calculated from the distance between the third toe and hind limb pads (print length) and the distance between the first and fifth toes (print width). Measurements of these parameters were obtained from the right (lesioned) and left (uninjured) paw prints, and the PFI was calculated using the following formula by Bain and colleagues [52]:

PFI = 174.9 × ((EPL - NPL)/NPL) + 80.3 × ((ETS - NTS)/NTS) – 13.4

where N is the normal or nonoperated side, E is the experimental or operated side, PL is the print length, and TS is the total toe spread or distance between the first and fifth toe. Further, in depth-analysis of step kinematic parameters was carried out. For that, parameters such as max contact area (cm^2^), step sequence - Regularity index, and base of support between front and hind paws, were studied. 

### Specimen preparation

One, four or twelve weeks after VRA, the animals were euthanized. Rats were anesthetized with a combination of xylazine (Anasedan, 10 mg/kg) and ketamine (Dopalen, 90 mg/kg). The vascular system was subjected to transcardial perfusion with phosphate-buffered saline (PBS) (pH 7.4). To evaluate neuronal survival and immunofluorescence, we perfused the rats with 4% paraformaldehyde in 0.1 M sodium phosphate buffer (PB) (pH 7.4). The lumbar intumescence was dissected out and post fixed in the same fixative for 12 h at 4°C. The specimens were then washed in PBS and subjected to gradually increasing concentrations of sucrose (10, 20 and 30% for 24 h each) in 0.1 M PB for cryoprotection. The samples were then embedded in Tissue-Tek O.C.T. (Sakura Finetek USA, Inc., Torrance, USA) and frozen at −35/40°C. Transverse sections of the corresponding L4-L6 spinal cord with 12 μm thick were collected on glass slides using a cryostat and subsequently stored at −20°C until use. For RT-PCR, the vascular system was subjected to transcardial perfusion with PBS (pH 7.4) only, and the spinal cord lumbar intumescence was dissected out, bisected (ipsi and contralateral sides) and frozen in liquid nitrogen.

### Motoneuron survival

Transverse spinal cord sections were stained in cresyl violet (Nissl stain) at room temperature. All motoneurons present in the lateral motor nucleus of the ventral horn on the ipsilateral (injured) and contralateral (uninjured) sides were counted in alternate sections from each specimen. Twenty sections 240 μm apart were used for counting in each specimen. The percentage of surviving cells was analyzed as a ratio of absolute numbers of motoneurons, counted per section, on the ipsilateral *versus* the contralateral side. The data are presented as the mean ± standard error of the mean (SEM) for each group.

### Immunofluorescence

The slides with the transverse sections of spinal cord were acclimatized, washed with 0.01 M PB (3 times for 5 min each) and incubated with 3% bovine serum albumin (BSA) in the same buffer for 1 h. The slides were then incubated for 3 h with primary antibodies (Table 1). The primary antibodies were diluted in a solution containing 1% BSA and 0.2% Triton X-100 in 0.1 M PB. Immunostaining was performed in a moist chamber at room temperature. After the sections were washed with 0.01 M PB (3 times for 5 min each), they were incubated with the secondary antibody according to the origin of the primary antibody diluted in 1% BSA and 0.2% Triton X-100 in 0.1 M PB for 45 min. The slides were washed again in 0.01 M PB, mounted in 0.1 M PB with glycerol + DAPI (3:1) and subsequently analyzed with a fluorescence microscope (Leica DM5500B) coupled with a Leica DFC345FX (Leica Microsystems CMS GmbH) camera. A rhodamine filter (CY2) was used to analyze the labeling of reactive astrogliosis (GFAP) and microglial reactions (IBA- 1) as well as synaptic vesicles (VGLUT-1 and GAD65). Representative images were captured from each side of the sections (both injured and uninjured) at magnification of 20x; therefore, three sections of the spinal cord ventral horn at lamina IX were used per experimental animal. For quantification, the integrated pixel density was measured in the entire picture, as previously described [53], by using the ImageJ software. The ratio of the integrated pixel density between the ipsilateral *versus* the contralateral sides was calculated for each animal. The data are represented as the mean ± standard error of the mean (SEM) for each group.

**Table 1.** Primary antibodies used for the immunofluorescence assay. Each antibody is followed by the supplier, host animal, product code, and concentration.

Antibody	Supplier	Host	Product code	Concentration
GFAP	Abcam	Rabbit	AB7779	1:1,500
IBA-1	Wako	Rabbit	019-19741	1:750
VGLUT-1	Synaptic Systems	Mouse	135303	1:1,000
GAD65	Abcam	Mouse	Ab26113	1:750
Alexa 488	Jackson Immuno Research	Anti-rabbit/Anti-mouse	711-545-152/715-545-150	1:800

### RT-qPCR

We evaluated the relative levels of mRNA for anti-and pro-inflammatory macrophage markers, anti-and pro-inflammatory cytokines, anti-and pro-apoptotic pathway proteins, and trophic factors (Table 2). Total RNA extracts were obtained from lumbar spinal cords one week after the VRA. The samples were mechanically homogenized/dissolved with a 200 μL pipette tip in Tryzol (QIAzol Lysis Reagent, Qiagen, Hilden, Germany), and the total RNA was extracted using the Tryzol protocol and RNeasy Lipid Tissue Mini Kit (Qiagen, cat nº 74804) according to the manufacturer's instructions. The quantification and quality of RNA samples were evaluated using a nanophotometer, and assessment of the RNA integrity was conducted by 1% agarose gel electrophoresis in denaturing conditions. Complementary DNA (cDNA) synthesis was performed using the *High Capacity cDNA Reverse Transcription Kit* (Applied Biosystems - 4368814) following the manufacturer's instructions. The cDNA was used in triplicate as a template for the PCRs in real time with TaqMan Gene Expression Master Mix (2x) (Life Technologies - PN 4369016) and TaqMan assays (primers + hydrolysis probes) for the genes described above, in a volume of 20 μL. Forty-five cycles of amplification were carried out (95°C for 10 min, followed by 95°C for 15 seconds and 60°C for 1 min). Reference genes were carefully chosen based on their unchanged expression under various experimental conditions. The reference gene (for medullary samples) was labeled with the VIC fluorophore and the target genes with the FAM fluorophore. Quantitative PCR was performed using the Mx3005P instrument (Agilent, Santa Clara, CA, USA), and the results were calculated using the MaxPro program (Agilent). Relative quantification of the genes of interest was performed using the 2^−ΔΔCt^ method [54].

**Table 2.** mRNA primers used for RT-qPCR followed by product code.

Primer	Product Code
VEGFA	Rn01511602_m1
BDNF	Rn02531967_s1
iNOS	Rn00561646_m1
CD38	Rn00565538_m1
Arginase-1	Rn00691090_m1
EGR2	Rn00586224_m1
TNF-α	Rn01525859_g1
IL-6	Rn01410330_m1
TGF-β	Rn00572010_m1
IL-4	Rn01456866_m1
IL-13	Rn00587615_m1
Bad	Rn00575519_m1
Bcl-2	Rn99999125_m1
GAPDH	Rn01775763_g1

### Statistical analysis

Data are presented as the mean ± SEM and were compared by one-way analysis of variance (one-way ANOVA) followed by Newman-Keuls *post hoc* test for Nissl staining analysis, and Tukey *post hoc* test for immunostaining and RT-qPCR, to evaluate differences among groups. Two-way analysis of variance (two-way ANOVA) followed by Mann-Whitney test was used to evaluate the Catwalk system results. GraphPad Prism software, version 7, was used for all analyses. Means were considered significantly different when *p < 0.05, **p < 0.01, ***p < 0.001 and ****p < 0.0001.

## Results

### DMF promotes neuronal survival, reduction of gliosis and synaptic preservation in a dose-dependent manner

*DMF promotes motoneuron survival*

**Figure 2.**
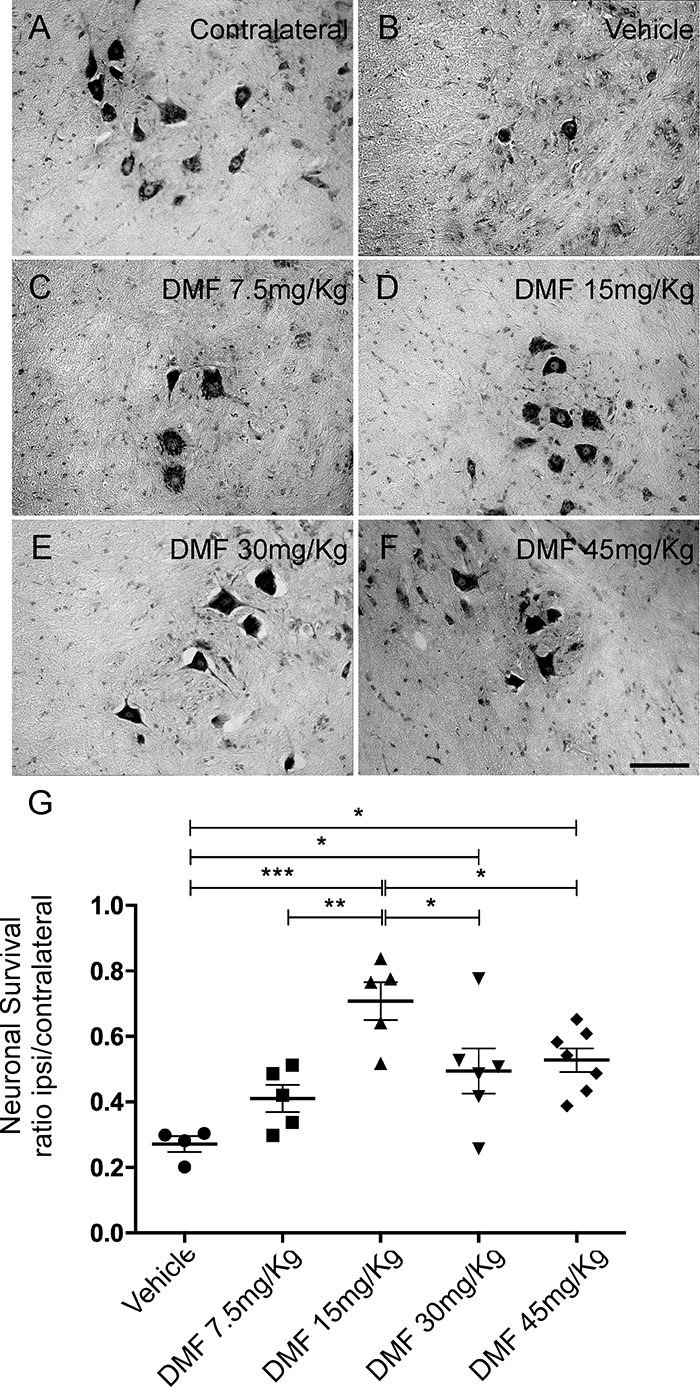
DMF treatment promoted motoneuron survival after VRA. **(** A **-** F **)** Nissl staining of transverse cross sections of the spinal cord at laminae IX in five different experimental groups 4 weeks following VRA. Note the decreased number of motoneurons ipsilateral to the lesion and the significant enhancement in neuronal survival in the group treated with 15 mg/kg DMF. **(** G **)** Average number of spared motoneurons present in the spinal cord 4 weeks after avulsion (***p < 0.001, **p < 0.01 and *p < 0.05). Scale bars = 200 μm. Mean ± SEM.

We observed 73% neuronal loss at 28 days after injury (Fig. 2B), which was reversed by DMF treatment. DMF at doses of 15, 30 and 45 mg/kg increased the rate of survival to 70% (***p < 0.001) (Fig. 2D), 54% (**p < 0.01) (Fig. 2E) and 52% (**p < 0.01) (Fig. 2F), respectively. The lowest dose, 7.5 mg/kg, was not able to achieve neuron survival, with a survival rate of 41% (Fig. 2C). However, even if treatment resulted in a higher survival rate, the motoneurons showed morphological changes, such as chromatolysis and nuclear displacement. The ratios of the number of surviving motoneurons in the ipsilateral to that in the contralateral sides were as follows - vehicle: 0.271 ± 0.023; 7.5 mg/kg DMF: 0.410 ± 0.041; 15 mg/kg DMF: 0.707 ± 0.057; 30 mg/kg DMF: 0.494 ± 0.069; and 45 mg/kg DMF: 0.527 ± 0.036 (Fig. 2G); F(4,22) = 8.479; p = 0.0003. Therefore, DMF treatment prevented neuronal death by increasing the percentage of surviving motoneurons in the spinal cord ventral horn, particularly at a dose of 15 mg/kg.

*DMF promotes a decrease in glial activity*

**Figure 3.**
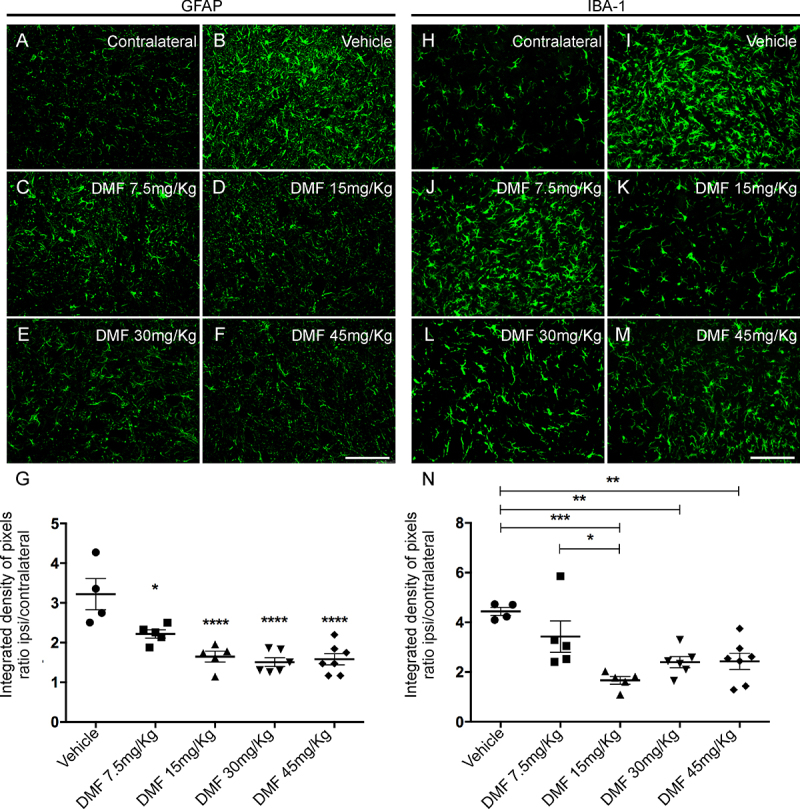
Immunofluorescence analysis of astrocytes and microglial reactions at the spinal cord lamina IX 4 weeks after avulsion using anti-glial fibrillary acid protein (GFAP) and anti-ionized calcium binding adaptor protein 1 (Iba-1) antibodies. **(** A **and** H **)** Contralateral sides. **(** B **-** F **)** Increased astrogliosis after VRA was observed on the ipsilateral side of the lesion. A significant reduction in astroglial immunoreactivity was observed in groups treated with 15, 30 and 45 mg/kg DMF but not 7.5 mg/kg DMF. **(** G **)** Ipsi/contralateral ratio of the integrated density of pixels of anti-GFAP antibody labeling in all groups (****p < 0.0001 and *p < 0.05). **(** I **-** M **)** Increased microgliosis after VRA was observed on the ipsilateral side of the lesion. A significant reduction in microglial immunoreactivity was observed in groups treated with DMF at 15, 30 and 45 mg/kg but not at 7.5 mg/kg. **(** N **)** Ipsi/contralateral ratio of the integrated density of pixels of anti-IBA-1 antibody labeling in all groups (***p < 0.001, **p < 0.01 and *p < 0.05). Scale bar = 100 μm. Mean ± SEM.

Astrocyte and microglial morphological reactivity were measured by immunolabeling with anti-GFAP and anti-IBA-1 antibodies, respectively and quantified by the integrated density of pixel ratios between the ipsi- and contralateral sides of each experimental group. After lesion, there was an increase in both astrocyte and microglial immunolabeling in the spinal cord ventral horn (Fig. 3B and 3I, respectively). After DMF treatment, astrogliosis was downregulated to levels significantly different from those in the lesioned counterpart (7.5 mg/kg: 0.0109; 15, 30 and 45 mg/kg: p < 0.0001) (Fig. 3C-F). DMF was able to decrease the microglial reaction as indicated by a decrease in Iba-1 labeling at all doses except the lowest (7.5 mg/kg: p = 0.3701; 15 mg/kg: p = 0.0004; 30 mg/kg: p = 0.0065; 45 mg/kg: p = 0.0057) (Fig. 3J-M). These results are directly related to the time point examined after lesion; the microglial reaction is typically enhanced during the acute post-lesion stage, and astrogliosis occurs later. The ratios of integrated GFAP-immunostained pixel densities in the ipsilateral side to those in the contralateral sides were as follows - vehicle: 3.220 ± 0.393; 7.5 mg/kg DMF: 2.219 ± 0.104; 15 mg/kg DMF: 1.647 ± 0.136; 30 mg/kg DMF: 1.508 ± 0.111; and 45 mg/kg DMF: 1.582 ± 0.141 (Fig. 3G); F(4, 22) = 14.08; p < 0.0001. For IBA-1-immunostained pixel densities were as follows - vehicle: 4.441 ± 0.162; 7.5 mg/kg DMF: 3.429 ± 0.629; 15 mg/kg DMF: 1.667 ± 0.156; 30 mg/kg DMF: 2.402 ± 0.223; and 45 mg/kg DMF: 2.432 ± 0.321 (Fig. 3N); F(4, 22) = 7.934; p = 0.0004.

*DMF promotes synaptic preservation*

**Figure 4.**
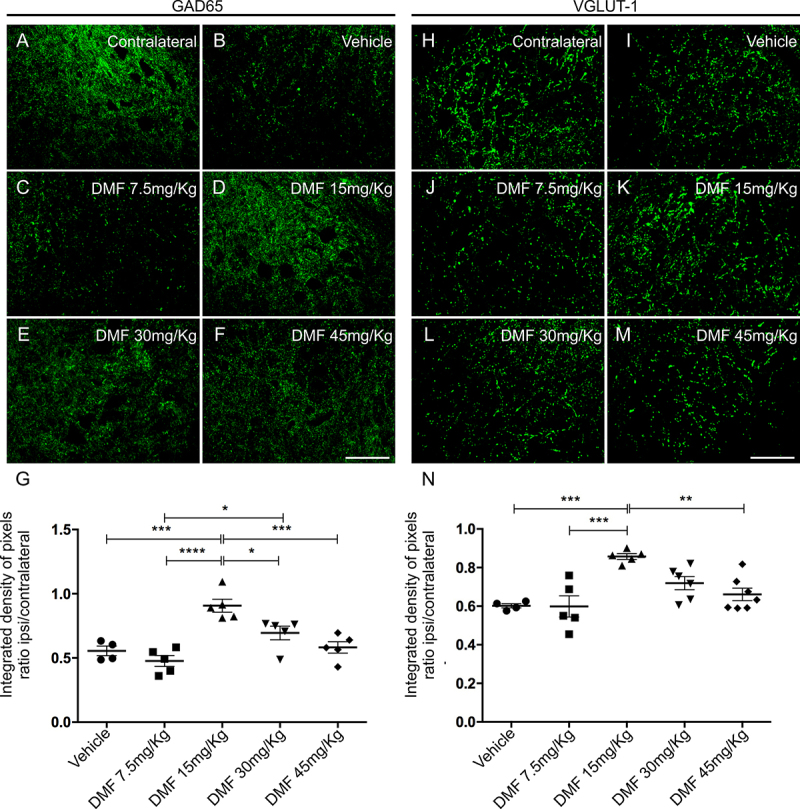
Immunolabeling of GABAergic and glutamatergic inputs at the spinal cord lamina IX 4 weeks after avulsion using anti-GAD65 (GABAergic inputs) and anti-VGLUT-1 (glutamatergic inputs) antibodies. **(** A **and** H **)** Contralateral sides. **(** B **-** F **)** Decreased GABAergic immunolabeling after VRA was observed on the ipsilateral side of the lesion. A significant augmentation of GABAergic immunoreactivity was observed in groups treated with 15, 30 and 45 mg/kg DMF but not 7.5 mg/kg. **(** G **)** Ipsi/contralateral ratio of the integrated density of pixels of anti-GAD65 antibody staining in all groups (****p < 0.0001, ***p < 0.001 and *p < 0.05). **(** I **-** M **)** Decreased glutamate immunolabeling after VRA was observed on the ipsilateral side of the lesion. Significant preservation of VGLUT-1 immunoreactivity was only observed in the group treated with 15 mg/kg DMF. **(** N **)** Ipsi/contralateral ratio of the integrated density of pixels of anti-VGLUT-1 immunofluorescence in all groups (***p < 0.001 and **p < 0.0.1). Scale bar = 100 µm. Mean ± SEM.

GABA and glutamatergic innervation were evaluated by immunolabeling for anti-GAD65 and anti-VGLUT-1 antibodies, respectively, using the integrated density of pixel ratios between the ipsi- and contralateral sides of each group. After lesion, there was an intense decrease in both inputs, with approximately 55% of GABAergic (Fig. 4B) and 60% of glutamatergic (Fig. 4I) inputs and terminals remaining in the spinal cord ventral horn ipsilateral to the VRA. After DMF treatment, we observed an indication of significant preservation of both synapse markers at the dose of 15 mg/kg, with 90% of GABAergic (p = 0.0005) (Fig. 4D) and 85% of glutamatergic (p = 0.001) (Fig. 4K) inputs and synapses preserved. The other doses, 7.5, 30 and 45 mg/kg, were not effective and provided similar results to the vehicle counterpart (Figs. 4C, E, F and 4J, L, M). The ratio of GAD65-immunostained integrated pixel densities in the ipsilateral to that in the contralateral side was as follows - vehicle: 0.554 ± 0,037; 7.5 mg/kg DMF: 0.476 ± 0.042; 15 mg/kg DMF: 0.907 ± 0.050; 30 mg/kg DMF: 0.694 ± 0.052; and 45 mg/kg DMF: 0.582 ± 0.044 (Fig. 4G); F(4, 19) = 13.13; p < 0.0001. For VGLUT-1-immunostained pixel densities were as follows - vehicle: 0.602 ± 0.010; 7.5 mg/kg DMF: 0.598 ± 0.054; 15 mg/kg DMF: 0.857 ± 0.014; 30 mg/kg DMF: 0.718 ± 0.034; and 45 mg/kg DMF: 0.660 ± 0.032 (Fig. 4N); F(4, 22) = 8.485; p = 0.0003.

### DMF associated with FS root reimplantation promotes neuronal survival, reduction of gliosis, excitatory synaptic preservation and motor function partial recovery

*DMF associated with root reimplantation and FS promotes neuronal survival*

**Figure 5.**
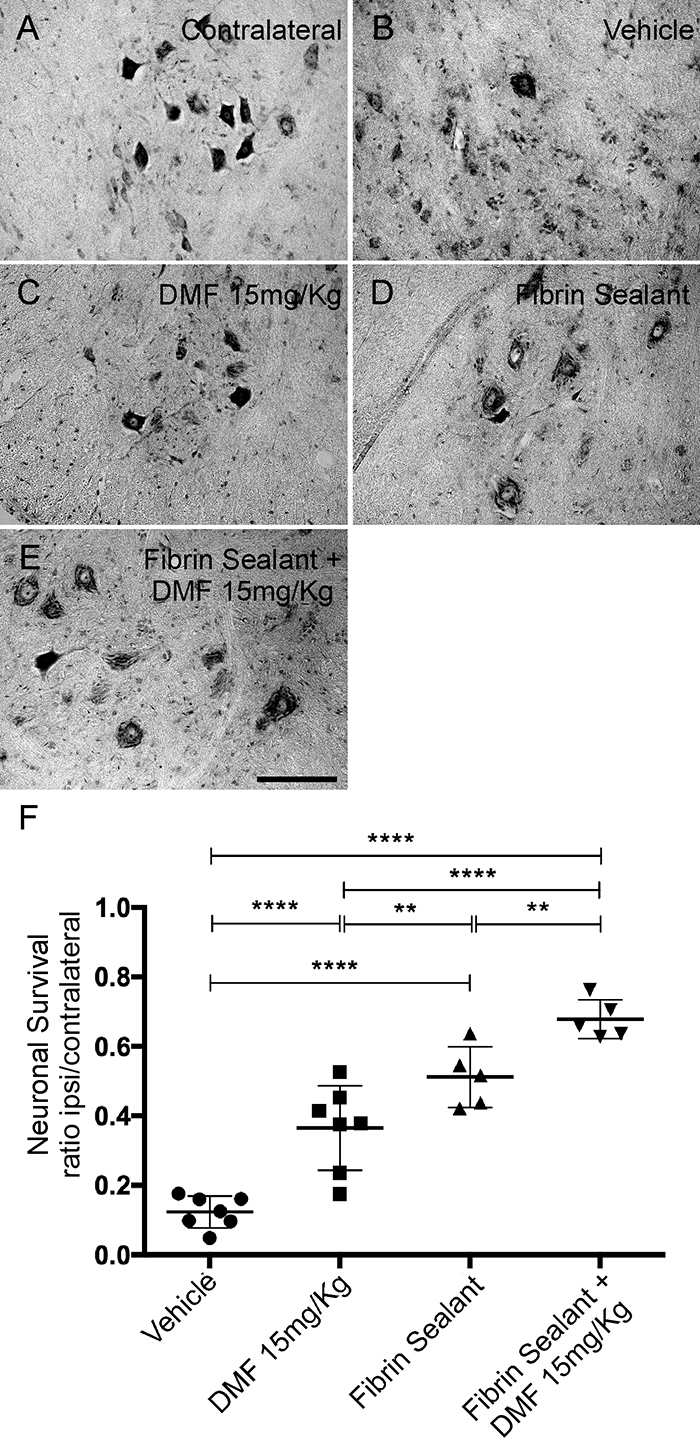
Motoneuron survival after VRA, root reimplantation and DMF treatment. **(** A **-** E **)** Nissl staining of transverse cross sections of the spinal cord at lamina IX in four different experimental groups 12 weeks after VRA. Note the decreased number of motoneurons ipsilateral to the lesion after VRA **(** B **)** and higher preservation when root reimplantation was performed with FS alone or in association with DMF treatment **(** D **and** E **)**. **(** F **)** Average number of spared motoneurons present in the spinal cord 12 weeks after avulsion (****p < 0.0001, ***p < 0.001 and *p < 0.05). Scale bar = 200 µm. Mean ± SEM.

We observed intense motor neuron degeneration 12 weeks after VRA, where only 12% of motoneurons survived the primary insult (Fig. 5B). After DMF treatment at 15 mg/kg, we observed an approximately 36% increase in motoneuron survival (Fig. 5C). For the groups in which root reimplantation was performed, the rate of neuronal survival was 51% with FS only (Fig. 5D) and 67% when FS was associated with 15 mg/kg DMF treatment (Fig. 5E). The ratio of the number of surviving motoneurons in the ipsilateral side to that in the contralateral side was as follows - VRA only: 0.124 ± 0.017;VRA + 15 mg/kg DMF: 0.365 ± 0.046; VRA + reimplantation + vehicle: 0.512 ± 0.039; and VRA + reimplantation + 15 mg/kg DMF: 0.678 ± 0.025 ; F(3, 20) = 45.67; p < 0.0001 (Fig. 5G).

*DMF associated with root reimplantation with FS promotes a decrease in glial activity*

**Figure 6.**
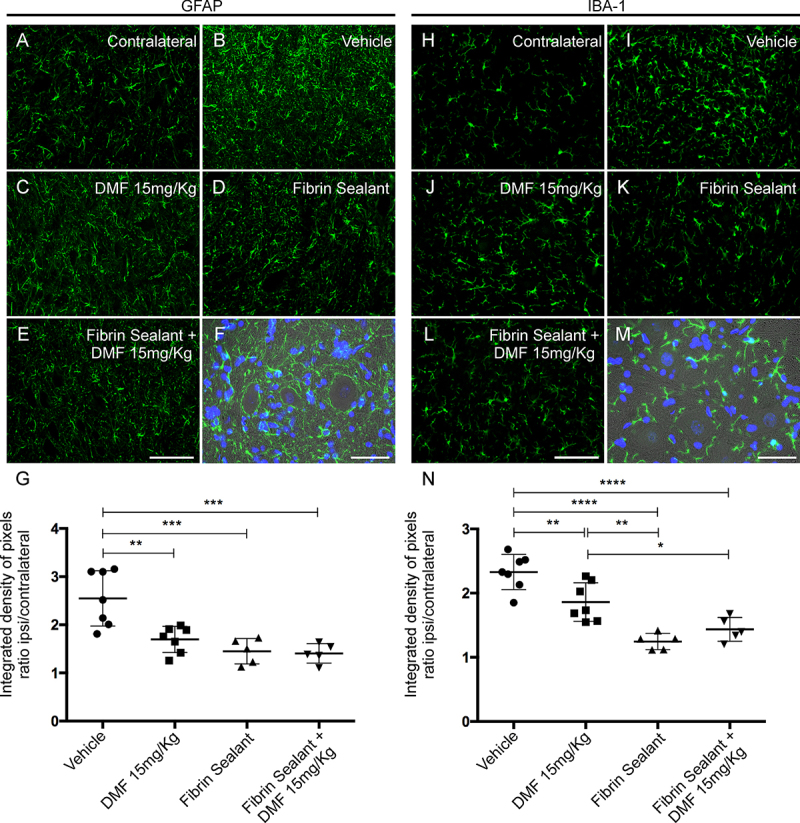
Immunofluorescence analysis of astrocytes and microglial reactions at the spinal cord lamina IX 12 weeks after avulsion using anti-glial fibrillary acid protein (GFAP) and anti-ionized calcium binding adaptor protein 1 (Iba-1) antibodies. **(** A **and** H **)** Contralateral sides. **(** B **-** E **)** Increased astrogliosis after VRA was observed on the ipsilateral side of the lesion. A significant reduction in astroglial immunoreactivity was observed in all groups with FS and/or 15 mg/kg DMF treatment. **(** G **)** Ipsi/contralateral ratio of the integrated pixel density of anti-GFAP antibody labeling in all groups (***p < 0.001 and **p < 0.01). **(** I **-** L **)** Increased microgliosis after VRA was observed on the ipsilateral side of the lesion. A significant reduction in microglial immunoreactivity was observed in all groups with FS and/or 15 mg/kg DMF. **(** N **)** Ipsi/contralateral ratio of the integrated pixel density of the anti-IBA-1 antibody in all groups (****p < 0.0001, **p < 0.01, *p < 0.05). Scale bar = 100 μm. Mean ± SEM. Double staining of **(** F **)** GFAP and **(** M **)** IBA-1 in green combined with DAPI (blue) and phase contrast surrounding large motoneurons in lamina IX. Scale bar = 50 μm.

Astrocyte and microglial morphological reactivity 12 weeks after VRA were measured by immunolabeling with anti-GFAP and anti-IBA-1 antibodies, respectively, and quantified by the integrated density of pixels ratio between the ipsi- and contralateral sides of each experimental group. After lesion, there was an increase in both astrocyte and microglial immunolabeling in the spinal cord ventral horn (Fig. 6B and 6I, respectively). After DMF 15 mg/kg (p = 0.0023), FS (p = 0.0004) and FS associated to 15 mg/kg DMF treatment (p = 0.0003), astrogliosis was downregulated to levels significantly different from those in the lesioned counterpart (Fig. 6C-E). Analysis of Iba-1 labeling demonstrated that 15 mg/kg DMF decreased the microglial reaction (p = 0.0094) (Fig. 6J). However, root reimplantation with FS showed a better response alone (p < 0.0001) or in combination with 15 mg/kg DMF (p < 0.0001) (Fig 6K and L, respectively). These effects were more moderate than those observed at 4 weeks after lesion because both astrocyte and microglial reactions are physiologically diminished at 12 weeks after lesion. Nevertheless, positive effects of both FS and DMF approaches were observed. The ratios of the GFAP-immunostained integrated pixel densities in the ipsilateral side to those in the contralateral side were as follows - VRA only: 2.550 ± 0.217; VRA + 15 mg/kg DMF: 1.697 ± 0.102; VRA + reimplantation + vehicle: 1.452 ± 0.118; and VRA + reimplantation + 15 mg/kg DMF: 1.407 ± 0.090; F(3, 20) = 12.42; p < 0.0001 (Fig. 6G), and those of IBA-1-immunostained densities were as follows - VRA only: 2.329 ± 0.103; VRA + 15 mg/kg DMF: 1.861 ± 0.113; VRA + reimplantation + vehicle: 1.246 ± 0.056; and VRA + reimplantation + 15 mg/kg DMF: 1.436 ± 0.082; F(3, 20) = 23.31; p < 0.0001 (Fig. 6N).

*DMF associated with root reimplantation with FS promotes synaptic preservation*

**Figure 7.**
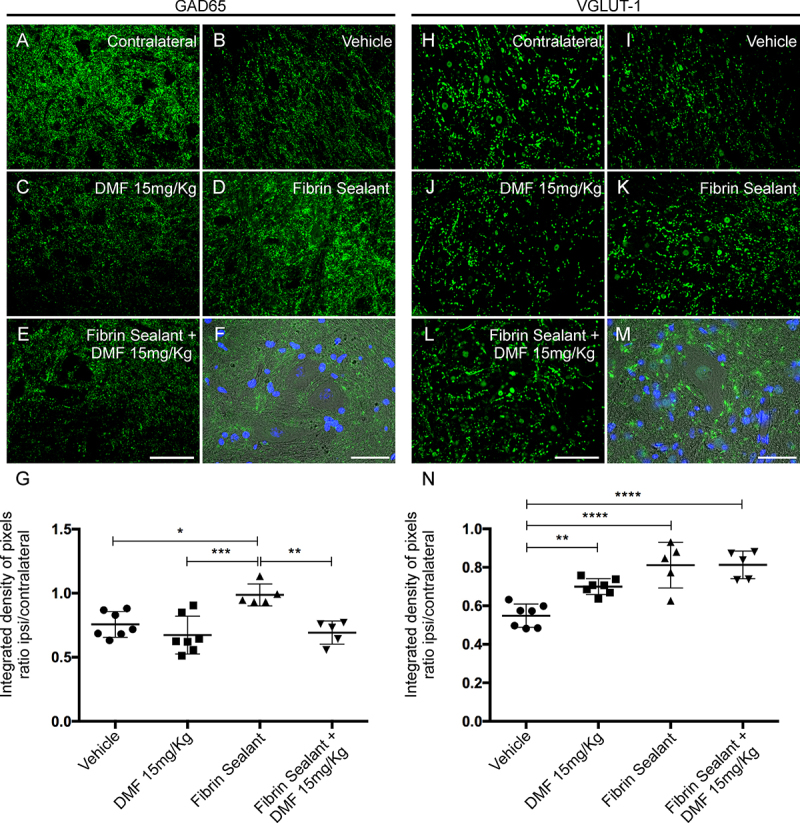
Immunolabeling of GABAergic and glutamatergic inputs at the spinal cord lamina IX 12 weeks after avulsion using anti-GAD65 (GABAergic inputs) and anti-VGLUT-1 (glutamatergic inputs) antibodies. **(** A **and** H **)** Contralateral sides. **(** B **-** E **)** Decreased GABAergic immunolabeling after VRA was observed on the ipsilateral side of the lesion. A significant augmentation of GABA immunoreactivity was observed after root reimplantation with FS but not with 15 mg/kg DMF. **(** G **)** Ipsi/contralateral ratio of the integrated density of pixels of the anti-GAD65 antibody in all groups (***p < 0.001, **p < 0.01, *p < 0.05). **(** I **-** L **)** Decreased glutamate immunolabeling after VRA was observed on the ipsilateral side of the lesion. A significant preservation of VGLUT-1 immunoreactivity was observed in the group treated with 15 mg/kg DMF and was greater when associated with root reimplantation. **(** N **)** Ipsi/contralateral ratio of the integrated density of pixels of the anti-VGLUT-1 antibody in all groups (****p < 0.0001, **p < 0.01). Scale bar = 100 μm. Mean ± SE. Double staining of **(** F **)** GAD65 and **(** M **)** VGLUT-1 in green combined with DAPI (blue) and phase contrast demonstrating the proximity of GABAergic and glutamatergic inputs to the motoneuron cell bodies. Scale bar = 50 μm.

GABA and glutamatergic innervation were evaluated by immunolabeling with anti-GAD65 and anti-VGLUT-1 antibodies, respectively, and quantified using the integrated pixel density ratio between the ipsi- and contralateral sides of each group. After lesion, there was an intense decrease in both inputs, with approximately 75% of GABAergic (Fig. 7B) and 54% of glutamatergic (Fig. 7I) terminals remaining in the spinal cord ventral horn ipsilateral to the VRA. After 15 mg/kg DMF, only glutamatergic inputs show indication of preservation (p = 0.0053) (Fig. 7J). Root reimplantation with FS was able to preserve both GABAergic (p = 0.0114) and glutamatergic inputs (p < 0.0001) (Fig. 7D and K). However, when associated with 15 mg/kg DMF, GABAergic inputs were diminished (p = 0.0027) when comparing to FS alone (Fig. 7E). The ratios of GAD65-immunostained integrated pixel densities in the ipsilateral side to that in the contralateral side were as follows - VRA only: 0.756 ± 0.037; VRA + 15 mg/kg DMF: 0.673 ± 0.055; VRA + reimplantation + vehicle: 0.989 ± 0.038; and VRA + reimplantation + 15 mg/kg DMF: 0.692 ± 0.040; F(3, 20) = 8.72; p = 0.0007 (Fig. 7G), and those of VGLUT-1-immunostained densities were as follows - VRA only: 0.548 ± 0.022; VRA + 15 mg/kg DMF: 0.699 ± 0.015; VRA + reimplantation + vehicle: 0.811 ± 0.052; and VRA + reimplantation + 15 mg/kg DMF: 0.812 ± 0.032; F93, 20) = 17.49; p < 0.0001 (Fig. 7N).

### DMF associated with FS root reimplantation promotes immunomodulation of cytokines and macrophage polarization (Table 3)

*Anti-inflammatory cytokines*

**Figure 8.**
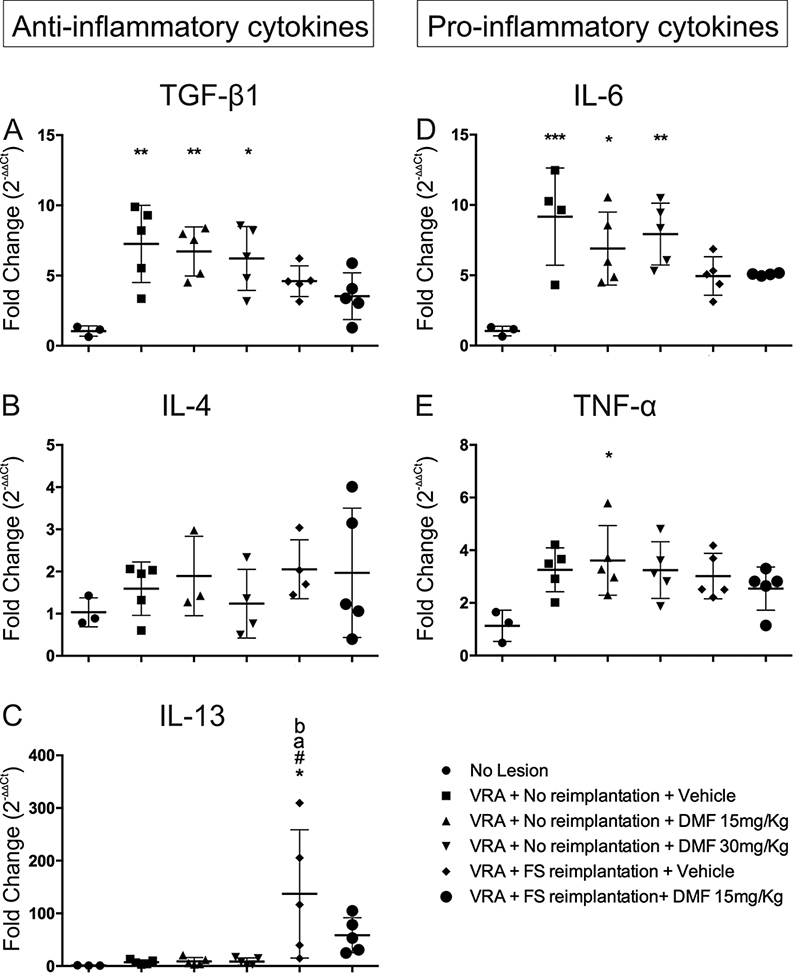
Relative expression of the anti-inflammatory cytokine genes TGF-β1, IL-4 and IL-13 and the pro-inflammatory cytokine genes IL-6 and TNF-α 7 days after injury. ***p < 0.001, **p < 0.01 and *p < 0.05 related to the uninjured group, **a** p < 0.05 compared to the 15 mg/kg DMF treatment group, and **b** p < 0,05 compared to the 30 mg/kg DMF treatment group. Mean ± SEM.

Gene transcripts for TGF-β1, characteristic of M2 macrophages related to inflammation, were upregulated in all experimental groups without root reimplantation when compared to those in the uninjured group; F(5, 22) = 5.835; p = 0.0014 (Fig. 8A). IL-4 expression showed no differences among groups; F(5, 18) = 0.6906; p = 0.637 (Fig. 8B). Only the group with root reimplantation showed a significant difference in IL-13 expression when compared to all other groups; F(5, 22) = 4.678; p = 0.0046 (Fig. 8C).

*Pro-inflammatory cytokines*

IL-6 gene transcript levels were higher for all non-reimplanted groups when compared to those in the uninjured group; F(5, 20) = 6.476; p = 0.0010 (Fig. 8D). Only the 15 mg/kg DMF group showed a significant upregulation in TNF-α expression when compared to the uninjured group; F(5, 22) = 2.941; p = 0.0351 (Fig. 8E).

*Macrophage subtypes*

**Figure 9.**
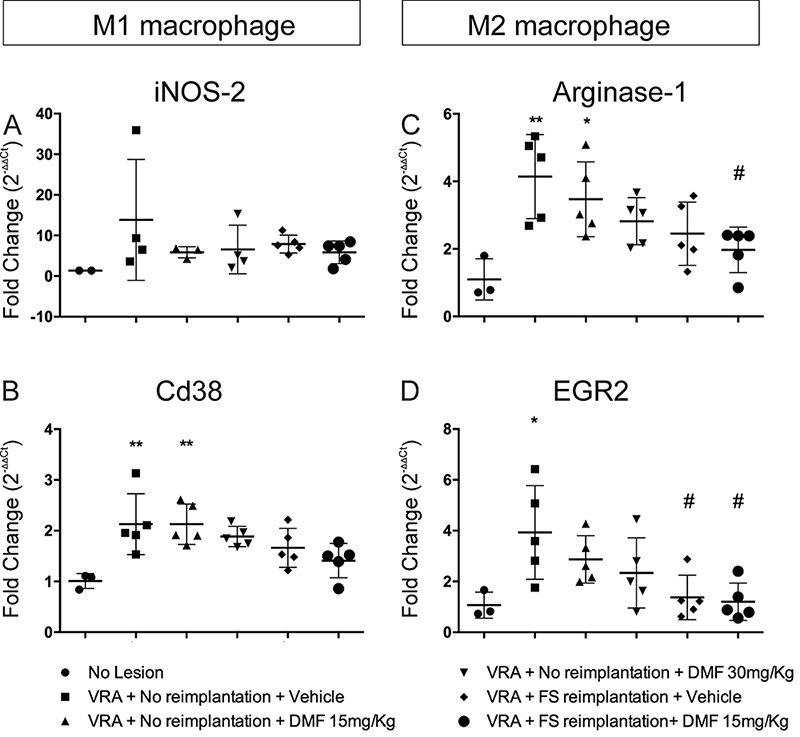
Relative expression of M1 macrophage-related genes iNOS-2 and CD38 and M2 macrophage-related genes arginase-1 and EGR2 7 days after injury. **p < 0.01 and *p < 0.05 compared to the uninjured group, and # p < 0.05 compared to the VRA only group. Mean ± SEM.

To determine the fate of macrophages present in the spinal cord seven days after injury, we assessed iNOS-2 and CD38 gene expression for M1 polarization (pro-inflammatory) and arginase-1 and EGR2 for M2 (anti-inflammatory) polarization. Although iNOS-2 transcript levels were not different among the groups [F(5, 17) = 1.077; p = 0.4075], CD38 transcript levels were higher in the VRA only group and in the group treated with 15 mg/kg DMF than in the uninjured group, with no differences among the other groups; F(5, 22) = 5.014; p = 0.0032 (Fig. 9A and B, respectively). Arginase-1 transcript levels were also higher in the VRA group and groups treated with 15 mg/kg DMF than in the uninjured group but also lower after root reimplantation associated with 15 mg/kg DMF treatment than after VRA alone; F(5, 22) = 5.519; p = 0.0019 (Fig. 9C). The VRA only group showed higher transcript levels of EGR2 than the uninjured group, and EGR2 levels were lower in both groups in which root reimplantation occurred than in the VRA only group; F(5, 22) = 4.262; p = 0.0073 (Fig. 9D). These results show that both M1 and M2 macrophages are present seven days after injury.

*Trophic factors*

**Figure 10.**
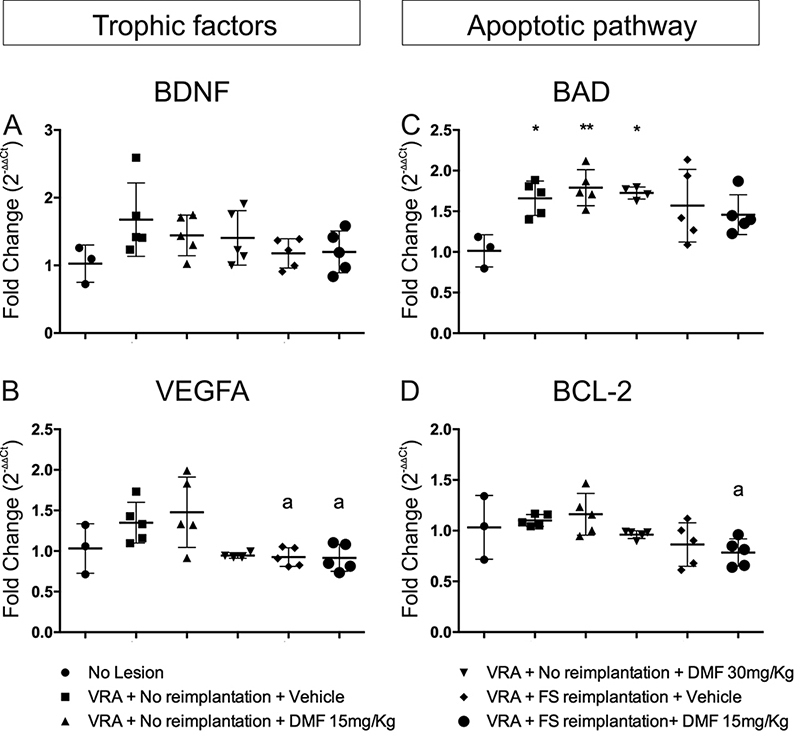
Relative expression of trophic factor genes BDNF and VEGFA and apoptotic pathway protein genes Bad (pro-apoptotic) and Bcl-2 (anti-apoptotic), 7 days after injury. **p < 0.01 and *p < 0.05 compared to the uninjured group, and **a** p < 0.05 compared to the 15 mg/kg DMF treatment group. Mean ± SEM.

The gene expression of the trophic factor BDNF showed no differences among groups; F(5, 22) = 1.772; p = 0.1603 (Fig. 10A). Trophic factor VEGFA, on the other hand, showed diminished transcript levels only in the reimplanted groups when compared to the 15 mg/kg DMF group; F(5, 21) = 4.475; p = 0.0062 (Fig. 10B).

*Apoptotic pathway*

Bad transcript levels were higher in all groups without root reimplantation than in the uninjured group; F(5, 21) = 3.820; p = 0.0128 (Fig. 10C). Only the group with root reimplantation associated with 15 mg/kg DMF exhibited reduced Bcl-2 transcript expression when compared to the 15 mg/kg DMF group; F(5, 22) = 3.487; p = 0.018 (Fig. 10D).

**Table 3.** Summary of gene expression following root reimplantation associated with DMF treatment as compared to avulsion alone.

Target	Results summary
Anti-and pro inflammatory cytokines	Balance between anti- and pro-inflammatory cytokines, favoring towards anti-inflammatory state.
Macrophages subtypes	M1 and M2 macrophage polarization, towards resolution of the inflammatory reaction.
Trophic factors	No difference
Apoptotic pathway	Favoring neuroprotection.

### Motor function recovery after root reimplantation with FS and DMF treatment

**Figure 11.**
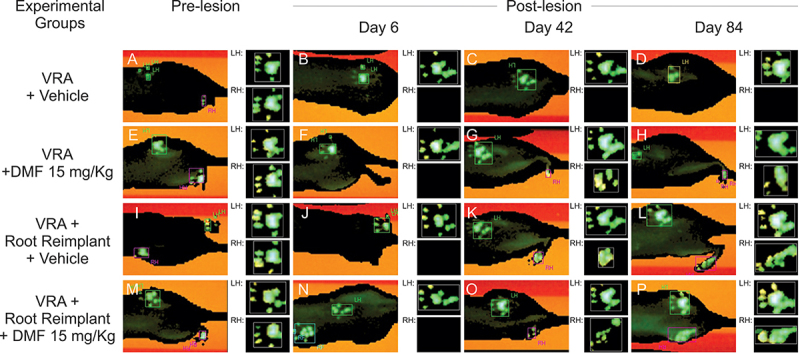
Right and left hind paw prints obtained with the Catwalk system at different time points showing the progressive motor function recovery after root reimplantation by FS and DMF treatment. **(** A **-** D **)** Animals submitted to VRA and treated with vehicle; note the loss of motor function. **(** E **-** H **)** Animals submitted to VRA and treated with 15 mg/kg DMF; animals showed minor motor recovery. **(** I **-** L **)** Animals submitted to VRA and root reimplantation and treated with vehicle; animals showed delayed motor recovery. **(** M **-** P **)** Animals submitted to VRA and root reimplantation and treated with 15 mg/kg DMF; animals showed substantial motor recovery.

**Figure 12.**
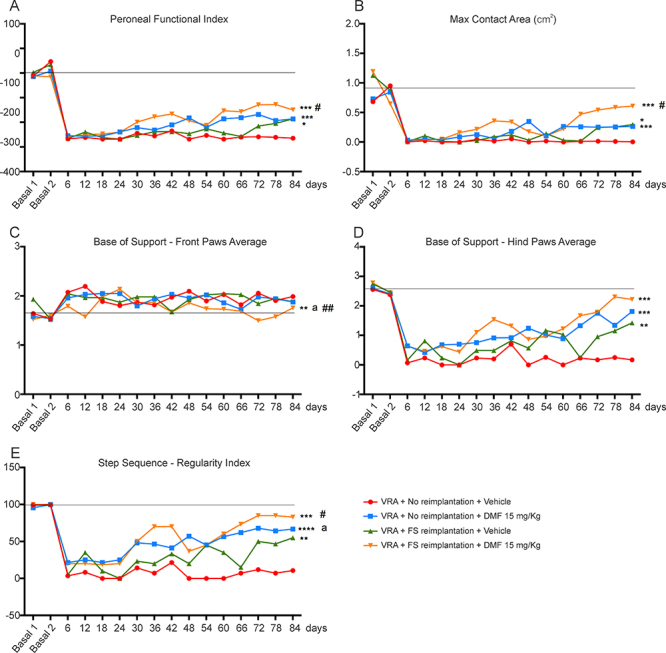
Catwalk gait analysis. **(** A **)** Peroneal nerve functional index, demonstrating functional improvement; root reimplantation and/or DMF treatment resulted in a significant improvement in performance compared to VRA only by day 84. Additionally, DMF treatment associated with root reimplantation showed a significantly earlier improvement (day 36) than the other treatments (p < 0.05). **(** B **)** Max contact at (cm^2^) demonstrating the maximum area of a paw that comes into contact with the glass plate. **(** C **)** Base of support - front paws average and **(** D **)** Base of support - hind paw average, showing that VRA does not alter the average width between front paws. As for hind paws, root reimplantation and/or DMF treatment resulted in restoration of normal width. **(** E **)** Step sequence - regularity index, showing improvements in inter-paw coordination after root reimplantation and/or DMF treatment, when compared to VRA and reimplantation. N = 5-7. ***p < 0.001, **p < 0.01 and *p < 0.05 compared to VRA group, ##p < 0.01 and #p < 0.05 compared to the reimplantation group, and **a** p < 0.05 compared to the 15 mg/kg DMF treatment group.

Motor function recovery is the result for effective neuronal survival and axon regeneration to the target muscles. A significant difference in the interaction between hindlimb print length and width given by the functional index of the peroneal nerve was observed among the groups with 15 mg/kg DMF treatment (p = 0.0007), reimplantation with FS (p = 0.0348) and FS associated to 15 mg/kg DMF treatment (p = 0.0006) (Figs. 11 and 12A). However, root reimplantation combined with DMF treatment showed considerable early improvements around day 36, while other groups showed delayed recovery. Therefore, the combination of pharmacological and surgical approaches was demonstrated to be significantly positive for functional recovery up to 12 weeks after injury. Differences between recovery curves were evaluated with the Mann-Whitney test, and *p < 0.05 for root reimplantation, **p < 0.01 for root reimplantation associated with 15 mg/kg DMF treatment and ***p < 0.001 for 15 mg/kg DMF treatment alone compared to VRA only. Two-way ANOVA revealed that DMF treatment associated with root reimplantation resulted in the earliest significant difference in the functional index 36 days after VRA (*p < 0.05), 12 days before DMF treatment alone exhibited a significant difference (*p < 0.05) and 48 days before root reimplantation alone (**p < 0.01). DMF treatment associated with root reimplantation also showed better results regarding the maximum area of the paw in contact with the Catwalk glass plate (p = 0.0002) (Fig. 12B). Regarding motor coordination, we observed that the base of support between paws are almost unaltered for front paws (Fig. 12C). After VRA, animals showed a great distance between hind paws, which were close to normal in all groups which received root repair and DMF treatment (Fig. 12D). As for step sequence, which is the order in which the paws are placed on the Catwalk glass plate, we observed that after VRA animals lack inter-paw coordination, contrarily to animals that received any of the treatments provided (Fig. 12E). It is important to emphasize that DMF treatment, when associated with root reimplantation showed close-to-normal hind limb coordination in contrast to VRA alone. 

## Discussion

VRA is a well-known severe injury that leads to a large loss of axotomized motoneurons [5-8], resulting in paralysis ipsilateral to the lesion. Motoneuron death following avulsion has been postulated to be influenced by the proximity of the axotomy to the cell body and due to the traction of the axon at the interface of the CNS and PNS. The cell death process is triggered by different events, including local ischemia, cell disruption with cytoplasmic leakage and local inflammation. Thus, the development of strategies to decrease the effects of such injury is necessary. In this sense, pharmacological therapy combined with avulsed root repair can mitigate neuronal loss and decrease the local inflammatory response, enhancing the success of the regenerative process.

In the present study, we observed survival of 30% of the lesioned motoneurons by the fourth week and 12% by the twelfth week after injury, with consequent loss of motor function, clinically identified as ipsilateral monoplegia. Importantly, chronic treatment with DMF showed robust neuroprotective activity, with 70% of the spinal motoneurons preserved four weeks after injury and 36% twelve weeks after. Although neuroprotective capacity has been observed in other experimental models of CNS diseases, the present work is the first to show DMF-induced neuroprotection following ventral root injury in the acute and chronic stages post injury. DMF has been shown to elevate GSH levels and the expression of antioxidant genes, such as HO-1, Mn-SOD and GPx (55, 56). 

We observed neuroprotection of 50% of lesioned motoneurons by 12 weeks after injury with root implantation using FS, a result that is in line with previous studies concerning ventral root reimplantation at the same time point [5, 6, 8]. The reconnection by itself allows motor axons to regrow towards the PNS [40, 57]. Additionally, reimplantation provides the glial-derived neurotrophic factors necessary for nerve regeneration [58-61].

Importantly, the combination of pharmacological support and anatomical root repair further enhanced motoneuron regeneration, allowing 70% of neurons to be preserved at the chronic stage post lesion. This finding is in contrast to the results from the group without the above-mentioned therapeutic approaches, where less than 30% of the neurons were preserved 12 weeks post lesion [6, 8]. Notably, DMF treatment was carried out and restricted to the first four weeks post injury, and the neuroprotective effects were maintained up to 12 weeks after injury. Taking into consideration the eventual side effects of prolonged DMF treatment, the present data indicate that a short period of treatment is sufficient to overcome the neuronal degeneration induced by VRA.

The present data on astrocyte and microglia activity after VRA are in line with the literature and previous works from our group [5, 6, 8]. Glial cells support neural functions, participate in the formation of the blood-brain barrier, perform phagocytosis of debris, influence the function of synapses, and are crucial for neuronal homeostasis processes [9, 53]. The activation of astrocytes and microglial cells in response to VRA, if pro-inflammatory, increases cell degeneration due to the release of toxic factors, inhibition of axonal growth and other degenerative processes [62]. Because these cells surround the alpha motoneurons, modulation of their reactivity is a key feature for achieving neuroprotection [63]. Microglia function as central macrophages, removing apoptotic or necrotic cell fragments and synaptic buttons. In contrast, astrocytes, in response to inflammatory stimuli, secrete chemokines that facilitate the recruitment of immune cells that infiltrate the CNS environment [64, 65].

Our results indicate that DMF treatment significantly decreases glial reactivity, as observed by diminished astrocytic and microglial immunolabeling, 4 and 12 weeks after VRA, demonstrating a possible immunomodulatory activity. A similar result was observed by Cordaro and colleagues [55], where such an effect was attributed to the ability of DMF to inhibit the IκBα complex kinase, a factor involved in the regulation of several genes responsible for the generation of mediators or proteins related to inflammation, such as IL-1β, TNF-α, iNOS and Cox-2 [55, 66]. 

Glial reactivity can be associated with loss of synapses and neuronal death [67, 68]. We observed a significant decrease in glial reactivity by DMF and root repair. Treatment with DMF was able to decrease both the microglia and astrocyte reaction, demonstrating a long-lasting effect observed 12 weeks post lesion, although DMF treatment was only administered for 4 weeks post injury. Similarly, the effects of nerve root reimplantation with FS led to a significant reduction in glial reactivity, as described in the literature [6, 8]. Thus, the reduction in astroglial and microglial reactivity may have contributed to the increase in neuronal survival (consistent with M1 x M2 modulation and BAD gene transcript downregulation observed herein) and synaptic preservation [6, 69-72].

Our data support the concept that a reduction in gliosis can be related to a better preservation of glutamatergic and GABAergic synapses, providing an overall preservation of spinal circuits, which facilitates regeneration and motor recovery. The connection between the glial reaction and the retraction of motoneuron inputs following axotomy is already known [67, 68]. In this sense, by reducing the glial reaction, the neuronal circuitry can be protected and improved. Accordingly, we observed an approximately 50% reduction in both glutamatergic and GABAergic presynaptic terminals in the ventral horn 4 weeks after VRA and between 25-40% reduction 12 weeks after VRA. On the other hand, treatment with 15 mg/kg DMF preserved at least 80% of the presynaptic terminals at both experimental time points. As previously mentioned, Jing and colleagues [35] also observed better preservation of the dopaminergic system and a reduction in gliosis upon DMF treatment in Parkinson’s disease. Moreover, Parodi and colleagues [73], in a multiple sclerosis model, observed that DMF treatment led to the induction of more action potentials, being able to normalize glutamatergic synapses in the CNS.

According to the literature, there is extensive loss of synapses 12 weeks after VRA, reducing the number of excitatory and inhibitory inputs by 60% [6, 8]. DMF treatment decreased overall synapse loss 12 weeks after VRA. Accordingly, reimplantation with FS also resulted in substantial synaptic preservation, mostly of inhibitory afferents to motoneurons [6, 8], positively influencing the recovery of motor coordination [40]. Therefore, the results support that early VRA repair stabilizes the spinal circuits, contributing to motor recovery. Importantly, when we performed DMF treatment and root reimplantation with FS, the balance of inhibitory and excitatory inputs seemed to be more prompt to the excitatory side, contrarily to the reimplantation alone. This might indicate that DMF treatment accelerated the regeneration process.

In addition to the abovementioned neuroprotective mechanisms, BDNF, GDNF and NT3 play an important role in survival and regeneration after CNS injury. Such neurotrophic molecules are directly related to axonal sprouting and elongation after peripheral nerve injury and during development [74]. Furthermore, Hallin and colleagues [59] suggested that the correction of motor deficits after ventral root avulsion and reimplantation in monkeys depends on the initial stimulation of CNS/PNS regeneration by neurotrophic factors and receptor expression [75]. Thus, Cordaro and colleagues [55] observed that DMF treatment after spinal cord injury increases the levels of such neurotrophic factors. In the present study, possibly due to the time point assessed (1 week after injury), we did not find upregulation of BDNF or VEGFA gene transcripts. Nevertheless, such an increase may have occurred at early stages post injury.

Animals who underwent root reimplantation combined with DMF administration demonstrated better motor performance after avulsion injury assessed by the walking track test, as well as different gait recovery parameters (inter-paw coordination, paw area, base of support and peroneal function recovery), similar to previous data in the literature [6, 8]. It is interesting to point out, however, that motor compensation events may also occur, since animals treated with DMF only, without root reimplantation, showed motor improvement. We believe that such motor compensation may be due to the partial preservation of the femoral (L3-L4) and obturator (L2-L4) nerves after avulsion [76]. 

Altogether, we observed that DMF and FS, alone or in combination, were able to facilitate nerve regeneration, leading to significant motor improvements. Such motor recovery was probably due to the plasticity of the CNS and PNS at different levels, including the motor cortex and ascending and descending pathways, as well as refinement of the motor units and a reduction in the inflammatory response mediated by the combined approaches [56]. 

## Conclusion

Treatment with DMF has robust neuroprotective and immunomodulatory effects following ventral root avulsion. Such effects included the preservation of motoneurons after injury, a decrease in glial reactivity and an increase in synaptic preservation at the lowest dose of 15 mg/kg. Additionally, the combination of DMF with FS and root repair improved neuronal survival, synaptic input preservation and immunomodulation and led to significant motor function recovery, which may in turn support a translation to the clinic. 
